# Ambient ozone exposure and children’s acute asthma in New York City: a case-crossover analysis

**DOI:** 10.1186/s12940-015-0010-2

**Published:** 2015-03-18

**Authors:** Perry Elizabeth Sheffield, Jiang Zhou, Jessie Loving Carr Shmool, Jane Ellen Clougherty

**Affiliations:** 1 Icahn School of Medicine at Mount Sinai, 1 Gustave L. Levy Pl., Box 1057, DPM, New York, NY 10029 USA; 2 Department of Environmental and Occupational Health, University of Pittsburgh Graduate School of Public Health, 100 Technology Drive, Pittsburgh, PA 15219 USA

**Keywords:** Air pollution, Ozone, Children, Asthma, Case-crossover, Time-series, Emergency department, Hospitalization

## Abstract

**Background:**

Childhood asthma morbidity has been associated with ambient ozone in case-crossover studies. Varying effects of ozone by child age and sex, however, have been less explored.

**Methods:**

This study evaluates associations between ozone exposure and asthma emergency department visits and hospitalizations among boys and girls aged 5-17 years in New York City for the 2005-2011 warm season period. Time-stratified case-crossover analysis was conducted and, for comparison, time-series analysis controlling for season, day-of-week, same-day and delayed effects of temperature and relative humidity were also performed.

**Results:**

We found associations between ambient ozone levels and childhood asthma emergency department visits and hospitalizations in New York City, although the relationships varied among boys and girls and by age group. For an increase of interquartile range (0.013 ppm) in ozone, there was a 2.9-8.4% increased risk for boys and 5.4-6.5% for girls in asthma emergency department visits; and 8.2% increased risk for girls in hospitalizations. Among girls, we observed stronger associations among older children (10-13 and 14-17 year age groups). We did not observe significant modification by age for boys. Boys exhibited a more prompt response (lag day 1) to ozone than did girls (lag day 3), but significant associations for girls were retained longer, through lag day 6.

**Conclusions:**

Our study indicates significant variance in associations between short-term ozone concentrations and asthma events by child sex and age. Differences in ozone response for boys and girls, before and after puberty, may point towards both social (gendered) and biological (sex-linked) sources of effect modification.

**Electronic supplementary material:**

The online version of this article (doi:10.1186/s12940-015-0010-2) contains supplementary material, which is available to authorized users.

## Background

As one of the six criteria air pollutants regulated by the U.S. Environmental Protection Agency (U.S. EPA), ozone still exceeds the National Ambient Air Quality Standard (NAAQS) in many U.S. urban areas, including New York City (NYC). Further, there is growing evidence of health effects occurring below the standard [1]. Ozone has been demonstrated in laboratory animals to cause increases in oxidative stress, epithelial injury and immunologic disease [2-5] and results from epidemiologic studies have found that short-term ozone exposure is associated with adverse health effects ranging from mild respiratory function impairment to increased mortality [6-10]. Effects of ozone on asthma outcomes have been examined widely, and significant associations between short-term ambient ozone exposures and asthma emergency department (ED) visits [11-15] and hospitalizations [16-18] have been reported, particularly during the warm season when ozone concentrations are higher [19,20].

According to the National Health Interview Survey Data from 2011, asthma is one of the most prevalent chronic conditions among U.S. children, with 10.5 million (14%) U.S. children having ever been diagnosed with asthma [21]. Children may be more susceptible to ambient air pollution than adults [22,23], and are disproportionately affected by asthma, as evidenced by higher asthma hospitalization rates for persons under age 18 [24]. Biological explanations for children’s heightened susceptibility to air pollutants may include higher ventilation rates, developing lung physiology, immature immune systems, and smaller peripheral airways, resulting in proportionally greater airway obstruction from inflammation [23,25-27]. Numerous epidemiologic studies have associated ozone with asthma exacerbations in children [28-31], but results have not always been consistent.

While children have been identified as a susceptible subpopulation, many of the prior studies of air pollution health effects have investigated associations across broad ranges of childhood ages, which may not reflect biologically meaningful periods for heightened sensitivity [32,33]. Differences in sex hormones, activity patterns, lung development, and immune systems between childhood and adolescence, which differ for boys and girls, would suggest that associations between air pollution and asthma could vary by both sex and age. Few epidemiologic studies, however, have been adequately powered to evaluate effect modification by age and sex groups simultaneously, potentially contributing to inconsistent findings among previous studies. In this study, we conducted case-crossover analysis to evaluate associations between ambient ozone concentration and asthma ED visits and hospitalizations among boys and girls aged 5-17 years in NYC, examining differential effects across sex and etiologically-relevant age groups. We compared case-crossover and time-series analytic methods, as a sensitivity test.

Prior ozone and asthma studies in NYC found that adjustment for other pollutants did not meaningfully change risk estimates. For example, Ito et al. [19] extensively examined the impact of model specifications on the risk estimates for asthma emergency department visits in NYC and found that inclusion of particulate matter less than 2.5 microns aerodynamic diameter (PM_2.5_), ozone and nitrogen dioxide (NO_2_) had little effect except that the risk estimates for sulfur dioxide (SO_2_) and CO were sensitive to the inclusion of NO_2_. In addition, in the evaluation of the role of age in modifying the effects of ozone and PM_2.5_ and asthma hospitalizations in NYC, Silverman and Ito [31] considered both single- and two-pollutant models, and found that adjustment for the other pollutant only slightly affected the risk estimates for these pollutants and that the age-specific effects pattern (i.e., the strongest association in the age 6-18 group) observed were not affected. Therefore, in this study we proceeded to focus on modification of ozone effects by sex and age in this same city.

## Methods

### Asthma case data

Ozone is a secondary pollutant formed through photochemical reaction of nitrogen oxides, volatile organic compounds, and sunlight, with notably higher concentrations during the warm season. Therefore, these analyses are limited to those months which correspond to the warm season in NYC. ED visits and hospitalizations have each been widely explored in response to short-term changes in air pollution exposures, but each captures a slightly different aspect of asthma exacerbation and use of medical services across sub-populations [34]. Hospital admissions are less frequent and thus may represent more severe cases, relative to outpatient events (wherein patients were treated then discharged) [35].

ED visit and hospitalization event data for asthma (ICD-9 code: 493) among children aged 5 to 17 years old in NYC from 2005-2011 (May 1-September 30) were obtained from the New York Statewide Planning and Research Cooperative System (SPARCS). We excluded case events for children younger than 5 years due to questionable reliability of asthma diagnosis among younger children [36]. Two categories of type of hospital admission - “emergent” (patient requires immediate medical intervention as a result of severe, life threatening, or potentially disabling conditions) and “urgent” (patient requires immediate attention for the care and treatment of a physical or mental disorder) - were selected to capture the acute effect of ozone. Patient data were grouped into these age groups: 5-9 years, 10-13 years, 14-17 years, and 5-17 years. Age groupings were selected, in part, based on the consideration of average age of onset of puberty [37,38]. The dataset was stratified by sex to compare associations for females and males.

### Ozone and other pollutant data from regulatory monitors

To construct a city-wide time series of ozone concentrations, hourly monitoring data was retrieved from the seven EPA Air Quality System (AQS) regulatory monitoring stations in NYC, for the years 2005-2011 (May 1 - September 30). We considered the spatial distribution of, and data density within, these regulatory monitors to prevent biasing the city-wide time trend. As two monitors in the Bronx were deployed at the same location and covered complementary data years (years 2005-2006 for Site ID 360050083, and 2007-2011 for Site ID 360050133), these were combined and treated as one monitor. Likewise, data from one monitor in Queens (Site ID: 360810098) was removed, as only one year of data was available. A city-wide time series was then derived from the five remaining monitoring locations, including data from four of five NYC boroughs - two in the Bronx (hereafter referred to as “Bronx A” and “Bronx B”), one in Manhattan, one in Queens, one in Staten Island. Descriptive statistics showed no systematic missing values by hour, day of week, month, or monitor. Hourly concentrations across the five monitoring sites correlated well over the entire time period (*r* = 0.76 to 0.95), supporting a consistent citywide temporal trend.

To evaluate and interpolate missing values to create a robust city-wide time-series, we assessed the proportion of missing values for each monitor – Bronx A = 1.4%, Bronx B = 26.9%, Queens = 5.5%, Manhattan = 36.8%, and Staten Island = 6.7%. We then used non-missing monitor values as the “independent” variable in successive linear regression models to interpolate missing values at each of the other monitors (dependent variables), starting with Bronx A because it had the fewest missing values (n = 348).

Because ozone has a diurnal pattern – with troughs in early morning (4-6 am) due to limited photochemical activity and scavenging by fresh vehicular emissions, and peaks in early afternoon (12-2 pm) - interpolation was conducted by hour and day of week, to minimize error in the mean trend across days. Third, daily average ozone values were computed from hourly values for each monitor, and a city-wide daily average ozone trend was computed by averaging daily values across all monitors. Because the measures from the two Bronx locations were highly correlated across all hours, their daily values were averaged to avoid over-weighting ozone measurements at this location.

For sensitivity analyses, daily co-pollutant time series for PM_2.5_ and NO_2_ were provided by the NYC Department of Health and Mental Hygiene. As with the ozone estimates, these data were calculated using data obtained from EPA AQS regulatory monitoring stations located in NYC (Zev Ross, unpublished report). The city-wide metrics were computed following an approach used by Schwartz [39] that uses averages of scaled daily values to account for differences in variance and mean between sites. The resulting city-wide time daily series was merged with the ozone time series described above.

### Weather data

Ambient daily temperature data from the four meteorological stations in the NYC area (JFK International Airport, LaGuardia Airport, Central Park, Newark International Airport) was retrieved from NOAA National Climatic Data Center (NCDC) [40]. Globally, minimum temperatures on land have increased more rapidly than maximum temperatures since 1950 (0.204°C/decade vs. 0.141°C/decade), resulting in a decline in the diurnal temperature range [41]. Physiologic recovery from daytime heat can be impaired if night time minimum temperatures are not sufficiently low [42]. In addition, a separate spatial pattern analysis of New York temperature data showed that minimum temperature has less spatial variation than maximum temperature and is generally representative of city-wide exposures (Zev Ross, unpublished data). Based on this reasoning, we chose to control for minimum temperature in the model. City-wide daily minimum temperature, mean temperature, and dew point temperature were averaged across the four stations. Relative humidity was calculated from mean temperature and dew point temperature using the standard NOAA equation [43].

### Statistical analysis

#### Case-crossover model

We used conditional logistic regression with time-stratified referent sampling in the case-crossover design [44]. Estimated risks were computed for interquartile-range (IQR) increments of ozone, and expressed as percent excess risk. Previous studies have reported increased asthma exacerbation event risk with elevated air pollution levels on current day and up to four days lag [10,45-47]. We estimated risks for lag days 0 to 6 to capture possible delayed health effects of ozone beyond the lag period commonly investigated. We evaluated differential effects across age and sex subgroups. Because case-crossover design allows for examination of effect modification at the level of the individual, rather than subgroup, we also modeled potential interactive effects between ozone, sex, and age.

#### Referent selection

A key issue in the case-crossover design is selection of referent time period, for which multiple strategies have been proposed, such as: unidirectional selection (referent times only prior to case event) [48], symmetric bidirectional selection (referent times both before and after event evenly) [49], and time-stratified selection (referent times within a given period, such as same-month) [50]. A systematic review on case-crossover analysis showed that only 7.7% used unidirectional design and the remainder bidirectional, most commonly using symmetric or time-stratified bidirectional designs (91.1% combined) [51]. Bi-directional time-stratified methods commonly select referent days to include non-case days on the same day of week, month, and year as the event, which can control for seasonal trends and day-of-week [52,53]. Accordingly, we defined case times as the day of an asthma hospitalization or ED visit, and matched with referent (control) days from the same day of week, in the same calendar month and year [44,54], thus controlling for day of week and season. Weather factors were controlled for by including smooth functions of: current-day minimum temperature (3 degrees of freedom [df]), lagged 6 day average minimum temperature (3 df), and relative humidity (3 df) in the model.

#### Sensitivity analyses

First, we conducted time-series analysis using Poisson regression to compare the results with the case-crossover model. The time-series analysis controlled for the same weather factors as the case-crossover model with same df, and also included a smooth function of time (8 df) per calendar year to control for long-term trends, and day of the week. In two separate multivariate case-crossover models, we substituted dew point for relative humidity and included co-pollutants – PM_2.5_ and NO_2_. To further examine counter intuitive negative associations observed within the case-crossover model (explained in ‘Results’ below), we created a 0-3 lag day average ozone variable to compare these results to the single day exposures. Furthermore, to explore ozone’s functional form, we: examined scatterplots of ozone versus daily asthma counts, stratified models by ozone concentrations and, ultimately, subset by month groupings. To avoid missing any delayed effect further than lag day 6, we conducted a lag days 7-9 analysis separately in time-series model but not in case-crossover model, as in our time stratified case-crossover design, any day beyond lag day 6 would qualify as a referent day.

Data were processed and analyzed in SAS version 9.3 (SAS Institute, Inc., Cary, North Carolina) and R statistical package (version 2.14.0; R Development Core Team 2013). This study was reviewed and approved by the Institutional Review Boards of the University of Pittsburgh and Icahn School of Medicine at Mount Sinai.

## Results

There were a total of 8,009 child asthma hospitalizations during the warm seasons (May-September) of 2005 to 2011, including 4,650 (58%) for males and 3,359 (42%) for females (Table 1). Among younger children, in age groups 5-9 years and 10-13 years, boys represented a higher proportion of asthma admissions (60 and 61%) than did girls (39 and 40%). Among older children (14-17 years), a higher proportion of admissions were for girls (54%) than for boys (46%). Over this same period, there were 35,907 asthma ED visits for 5-17 year olds, including 21,047 (58.6%) for males and 14,860 (41.4%) for females (Table 1). Similarly, the proportion was higher for boys than for girls among younger children (62 vs. 38% for both age subgroups), and higher for girls among older children (54 vs. 46%). Daily ozone values during our study period ranged from 0.005 ppm to 0.06 ppm (IQR 0.013 ppm).

**Table 1:** **Asthma events for NYC children in the warm months from 2005 to 2011**

Category	Male (percentage)	Female (percentage)	Total
Hospitalization			
Age 5-9 y	2646 (61)	1710 (39)	4356
Age 10-13 y	1376 (60)	907 (40)	2283
Age 14-17 y	628 (46)	742 (54)	1370
Age 5-17 y	4650 (58)	3359 (42)	8009
Emergency department visit			
Age 5-9 y	11145 (62)	6748 (38)	17893
Age 10-13 y	6466 (62)	4016 (38)	10482
Age 14-17 y	3436 (46)	4096 (54)	7532
Age 5-17 y	21047 (59)	14860 (41)	35907

### ED visits

**Figure 1:**
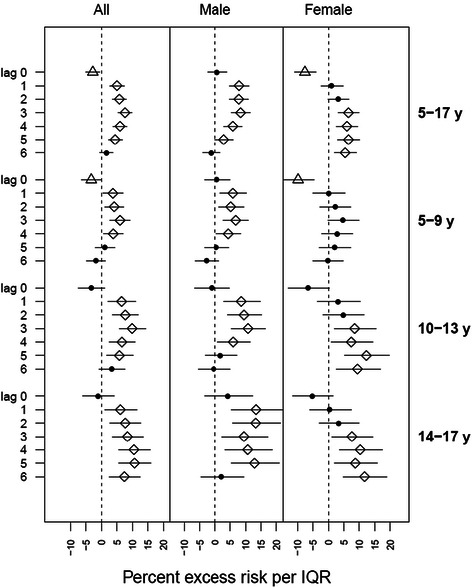
**Percent excess asthma emergency department visit risk in NYC children per interquartile range of ozone concentration.** Point denotes non-significant association; diamond denotes positive significant association; triangle denotes negative significant association.

The percent excess risk of asthma ED visits with higher ozone was significant for both males and females on multiple lag days in the all-ages analysis (5-17 years) (Figure 1); excess risk per IQR increase in ozone ranged from 2.9% (95% CI: 0, 5.9%) to 8.4% (95% CI: 5.4%, 11.5%) for boys, and from 5.4% (95% CI: 2%, 9%) to 6.5% (95% CI: 3.1%, 10%) for girls. The lag times for the ozone effect appeared different for boys versus girls. For boys, the association showed a “U”-shaped lag distribution; no effect at lag day 0, peaking at lag day 3, followed by decreasing magnitude of association and no effect again at lag day 6. For girls, the association with ozone started at lag day 3, and persisted through lag day 6. We observed negative associations with same-day ozone (i.e., lag day 0) among girls, but not among boys.

Sex-and-age-stratified analysis revealed slight differences in significance and magnitude of associations across subgroups. The highest excess risk for asthma ED visits was found among boys aged 14-17 years on lag day 1 [13.4% (95% CI: 5.4%, 22%)]. For age group 5-9 years, associations appeared on lag day 1 and persisted through lag day 4 for boys; for girls no positive significant association was observed, and a negative association on lag day 0 was observed (but became non-significant when substituting the 0-3 lag day average ozone). For ages 10-13 years, associations started on lag day 1 and persisted through lag day 4 for boys; while for girls significant associations were observed from lag days 3 through 6. Associations for age group 14-17 years showed a similar pattern as age group 10-13 years; significant associations for girls started about 2 days later than for boys, but lasted through lag day 6, when associations for boys became non-significant.

### Hospital admissions

**Figure 2:**
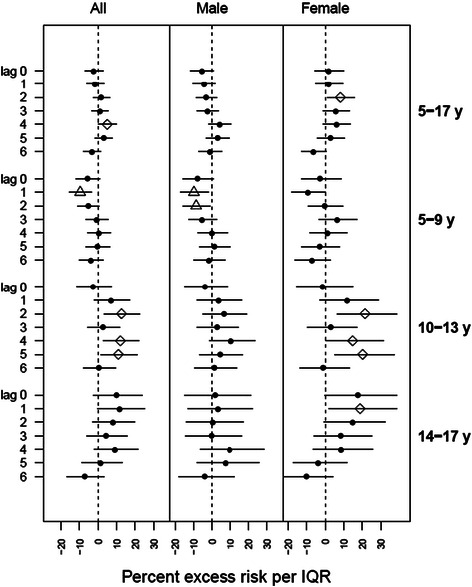
**Percent excess asthma hospitalization risk in NYC children per interquartile range of ozone concentration.** Point denotes non-significant association; diamond denotes positive significant association; triangle denotes negative significant association.

The risk of asthma hospitalization was significantly associated with ozone only for girls (5-17 years) (Figure 2); for an IQR increase in ozone, we observed significant excess risk of 8.2% (95% CI: 1.1%, 15.8%) on lag day 2. In analyses stratified by both age and sex, negative associations were observed for boys in age group 5-9 years on lag days 1 and 2. Significant associations were observed among girls aged 10-17 years. The highest excess risk for asthma hospitalization was found among girls aged 10-13 years on lag day 2 [21.5% (95% CI: 6.6%, 38.6%)].

### Sensitivity analyses

Results from time-series analysis for childhood asthma ED visits and hospitalization are shown in Additional file 1: Figure S1 and Additional file 2: Figure S2, respectively. The associations and the lag structure patterns are generally consistent with the main case-crossover results. In a case-crossover model testing interaction terms for sex and age (results not shown), the Wald *chi-square* test indicated statistically significant modification of ozone effects by both age and sex, with associations very similar to those revealed in the main case-crossover analysis. Time-series analysis for lag days 7-9 showed that the associations dropped to non-significant levels on lag day 7 for all age groups (results not shown). The results of the sensitivity analyses substituting dew point for relative humidity and including PM_2.5_ and NO_2_ were essentially identical to the initial model. The counter-intuitive negative associations above became positive or non-significant when we substituted the 0-3 lag day average ozone for these ED visit groups: all 5-17 years [9.3% (95% CI: 5.9%, 12.9%)]; all 5-9 years [6.4% (CI 95% 1.7%, 11.4%)]; females 5-17 years [2.6% (-2.3%, 7.8%)]; and females 5-9 years [-0.5% (95% CI -7.5%, 7%)]. However, the negative associations persisted even when using the 0-3 lag day average ozone levels among the hospitalizations for those noted above. Stratifying by ozone level did not show a consistent pattern that differed by ozone level. However, subsetting by month groupings (May-July and August-September) revealed that the observed negative associations were driven primarily by these latter months. Possible explanations for this temporal phenomenon are discussed below.

## Discussion

The boys had a higher proportion of asthma ED visits and hospital admissions than girls in the 5-13 year age group; the pattern reversed in the 14-17 year age group where girls had a higher proportion of asthma events, a pattern that is similar to those reported elsewhere [55-58]. Ozone levels were significantly associated with ED visits and hospitalizations for childhood asthma in NYC, although relationships varied between boys and girls. For girls, the strongest ozone and asthma ED visit association occurred in the 10-13 and 14-17 year old groups, whereas no significant modification by age was observed for boys. The boys exhibited more prompt response (lag day 1) to ozone, compared to girls (lag day 3), but the significant associations for girls remained longer, through lag day 6.

Differing patterns in sex-stratified results are of particular interest. A number of hypotheses have been proposed to explain this variation in asthma prevalence and response to pollution by age in boys and girls, including sex-linked biological differences in lung and airway size and growth rates [59], sex hormones [60], or to gender-linked variation in chemical co-exposures including cosmetic products, cigarette smoking, activity patterns, and obesity [61,62]. Our dataset lacked body mass index and personal behavior or product use data, thus we could not explore obesity or co-exposures as possible effect modifiers.

In the sex-stratified results of ED visit (Figure 1), we did not observe significant modification by age for boys. For girls, however, we found multiple significant associations in age groups of 10-13 and 14-17 years. This greater asthma exacerbation effect of ozone observed in older girls (10-17 years) compared with younger ones (5-9 years) likely concurs with active puberty for girls. This finding is consistent in general with a previous study that demonstrated age and sex-related differences in the risk of asthma hospital admission, with the risk among boys higher than girls <9 years of age, and reversed after puberty (10-19 years) [63].

No positive significant association was found on lag day 0 for either sex group, which may be expected, as asthma response may be progressive over subsequent days, or there may be significant delays in reaching medical care. The lagged response to ozone exposure was earlier for boys (lag day 1) than girls (lag day 3) in age groups of 10-13 and 14-17 years, suggesting more rapid ozone response for boys compared to girls of the same age groups. There are some possible explanations for this finding. Boys have smaller airways relative to lung volume than girls, and thus may be more susceptible to air pollutants [64], or differences in smooth muscle and vascular functions, and hormonal status may influence ozone response [65-67]. A Canadian study [33] found shorter lags between nitrogen dioxide exposures and asthma hospitalization among boys (2 to 3 days) than girls (6 to 7 days), but no associations for ozone. The authors proposed that boys have smaller airways relative to lung volume than girls, and thus may be more susceptible to specific stimuli. Lin et al.’s study only analyzed children aged 6-12 years, but nevertheless supports our general finding. An asthma cohort study in Southern California observed that, in that cohort, girls were less likely to play team sports and spent less time outdoors than boys [68], possibly contributing to differences in ozone exposure misclassification and observed associations with asthma outcomes. As outdoor ozone concentrations are higher than indoors, combined with higher ventilation rates during exercise, differences in activity patterns may partially account for differences in observed associations between ambient ozone and asthma.

Our finding of delayed ozone response among girls remaining significant through lag day 6 highlights that the health effects of ozone may be underestimated by only examining through 3 to 4 lag days, commonly done in previous studies. For example, one pediatric asthma study reported the strongest association between ozone and asthma ED visits on lag day 4 among 5-12 years age group [32]; another study conducted in Helsinki, Finland reported significant associations between ozone exposure and asthma ED visits for children (<15 years) from lag day 0 through lag day 3, and an excess risk estimate of 24.6% (95% CI: 21.2%, 67.8%) for lag days 0-4 average [10]; but neither study explored further lag day analysis. Silverman and Ito also examined the association of ozone with acute asthma in NYC and found significant associations for children of ages 6-18 years through lag day 3 but didn't stratify by sex [31].

We found fewer significant associations for asthma hospitalizations than ED visits (Figure 2). A significant association was observed only for girls on lag day 2 (5-17 years) for hospitalizations. When analyzed by age group, multiple positive significant associations were found for girls in age groups of 10-13 and 14-17 years, but not for boys. A study in Helsinki, Finland found stronger associations between ozone and childhood ED visits for asthma than did prior studies of asthma hospitalizations, and the authors suggested that ED visit data may be more sensitive than hospitalization data for capturing asthma exacerbations [10]. Another possible explanation is our time-stratified design which selects referent times before or after case time, or both, and thus could be considered “partially” bidirectional and assumes that cases are still at risk after an event [69]. Sampling post-event referent times for the subjects who stayed hospitalized longer than 7 days, thus, could bias results, as the referent exposures after the case day do not reflect actual exposures and risk of hospitalization for subjects not yet discharged. Although it has been shown that in case-crossover design, the bias resulting from sampling post-event referent times is small [54], we cannot rule out this possibility. This finding should also be interpreted with caution given the smaller statistical power with fewer hospitalization events. The age-stratified hospitalization analyses enabled a more specific consideration of ozone effects for key age ranges of interest but were somewhat limited in statistical power.

Interestingly, our results also initially suggested a “protective” effect of ozone. These largely disappeared when we considered the 0-3 lag day averages among ED visits, but not for hospitalizations. When interpreting this finding, it is important to note that the hospitalizations are largely a subset of the ED visits (i.e. a small percentage of the ED visits go on to be hospitalized), which could be altering the observed ozone association. However, negative associations have also been observed in previous studies; Paulu and Smith, for example, reported negative associations between asthma-related ED visits and ozone concentrations in one year (2003) of their study period (2000-2003). The authors found neither marked differences in source data between 2003 and the prior years, nor indication of confounding strong enough to change the direction of the association. An Israeli study also found negative correlations between emergency room visits and same-day ozone concentrations [70]. Buchdahl and colleagues observed a U-shaped relation between ozone and asthma incidence, indicating that ozone concentrations below a critical level were associated with higher incidence [71], and suggested confounding or possible protective effect of mid-level ozone concentrations. In our study, one plausible explanation is exposure misclassification due to intra-urban variability in ozone levels, not captured in AQS data, or strong inverse spatial correlations between ozone and combustion-related primary pollutants in NYC [72].

Furthermore, some researchers have proposed alternate modeling techniques to remove this physiologically counter-intuitive finding [73,74]. Our additional sensitivity tests revealed that the latter months (August-September versus May – July) show different ozone and asthma outcome relationships. We hypothesize that this finding may be due to declining ozone concentrations during these months with increasing asthma outcomes (as asthma ED visits and hospitalizations peak often in the fall due to rhinovirus infection seasonality and the start of the school year). The inability to control for this periodicity in asthma exacerbations – distinct from longer-term seasonal trends – is a limitation of our time-stratified case-crossover design referent sampling scheme (i.e., the same day of week of the same month in the same year). That is, for the same-day of the week in these latter months, asthma ED visits in the latter half of the month tend to be higher than those in the earlier half; and declining ozone concentrations over the month show the opposite pattern, yielding a negative ozone-asthma relationship. This finding led us to conclude that the residual confounding of the within-season temporal patterns in ozone and asthma exacerbations appears to act as a primary driver of our observed counterintuitive negative associations.

A key strength of our analysis is the confirmatory results between case-crossover and time-series models. Our main case-crossover model demonstrated significant associations between ambient ozone and childhood asthma events, and the time-series model and the case-crossover model with interaction terms provided comparable results, suggesting that associations were reasonably robust. This agreement between analytic techniques echoes previous comparison studies. A recent study of multiple air pollutants on cardiac arrests, using both time-series and case-crossover designs, found that risk estimates in case-crossover results were slightly less significant [75]; the authors argued that a possible explanation is that the case-crossover method applied 12 degrees of freedom per year, compared with 7 degrees of freedom per year for the time-series design. A cohort study conducted in five European cities compared time-series, case-crossover, and extended Cox regression designs, generating similar results, and the authors concluded that case-crossover analyses might underestimate the strength of associations [76]. We did observe such a pattern that the risk estimates were slightly smaller for case-crossover results than time-series results from lag days 1 to 3, but slightly larger for case-crossover results from lag days 4 to 6.

There are several important limitations to our study. First, the use of central site monitoring locations to estimate ozone concentrations likely produces measurement error; assuming this error is non-differential, it may bias effect estimates towards the null. Further, it has been noted that ozone has considerable small-scale spatial variations within urban areas [77], and thus better study designs would capture this spatial variation as well. Carracedo-Martínez pointed out in a systematic review that the potential advantages of incorporating individual-level information in case-crossover design has not been fully explored [51]. Further, our study period was only 35 months, offering a relatively small count of asthma hospitalizations, relative to longer-term studies. Pollen could be a contributor of asthma burden, however, the daily counts were not available for this study. The peak pollen times in this geographical area are spring (trees – a time period not included in our study), June (grasses) and September (weeds). Given the highly time specific and relatively short duration of pollen peaks, we would posit that omission of pollen might bias toward the null. Despite these limitations, our study reasonably corroborates prior results, and contributes towards better understanding differential effects of ozone on childhood asthma exacerbations by sex and age.

## Conclusions

Our study showed that short-term ozone exposures in an urban U.S. setting were significantly associated with asthma-related ED visits and hospitalizations in children aged 5-17 years. Our results are generally consistent with a growing body of evidence demonstrating an association between short-term ozone exposures and asthma ED visits and hospitalizations, but revealed substantial variation in effects by sex and age. There was a pattern of a strong ozone-ED visits association for females aged 10 to 17, whereas males exhibited significant associations among all age groups, suggesting that pubertal onset may play a differential role in asthma exacerbation among males and females. The lag time for induction of asthma appeared to be shorter among boys than girls, which may be related to a combination of sex-linked biological differences and gender differences in behaviors and exposure patterns.

## Additional files

Additional file 1: Figure S1. Results from time-series analysis for child asthma emergency department visits as percent excess asthma risk in NYC children per interquartile range of ozone concentration. Point denotes non−significant association; diamond denotes positive significant association; triangle denotes negative significant association.
Additional file 2: Figure S2. Results from time-series analysis for child asthma hospitalizations as percent excess asthma risk in NYC children per interquartile range of ozone concentration. Point denotes non−significant association; diamond denotes positive significant association; triangle denotes negative significant association.

## Abbreviations

AQS: Air quality system
CDC: Centers for Disease Control and Prevention
CI: Confidence interval
ED: Emergency department
ICD: International Classification of Diseases
IQR: Inter-quartile range
NAAQS: National Ambient Air Quality Standard
NCDC: National Climatic Data Center
NOAA: National Oceanic and Atmospheric Administration
NYC: New York City
RH: Relative humidity
SPARCS: New York Statewide Planning and Research Cooperative System
U.S. EPA: United States Environmental Protection Agency
