# Supplementary figures and images for: Ambient ozone exposure and children’s acute asthma in New York City: a case-crossover analysis
Source: Environ Health. 2015 Mar 18;14:25. doi: 10.1186/s12940-015-0010-2 (PMC4373115; doi:10.1186/s12940-015-0010-2)

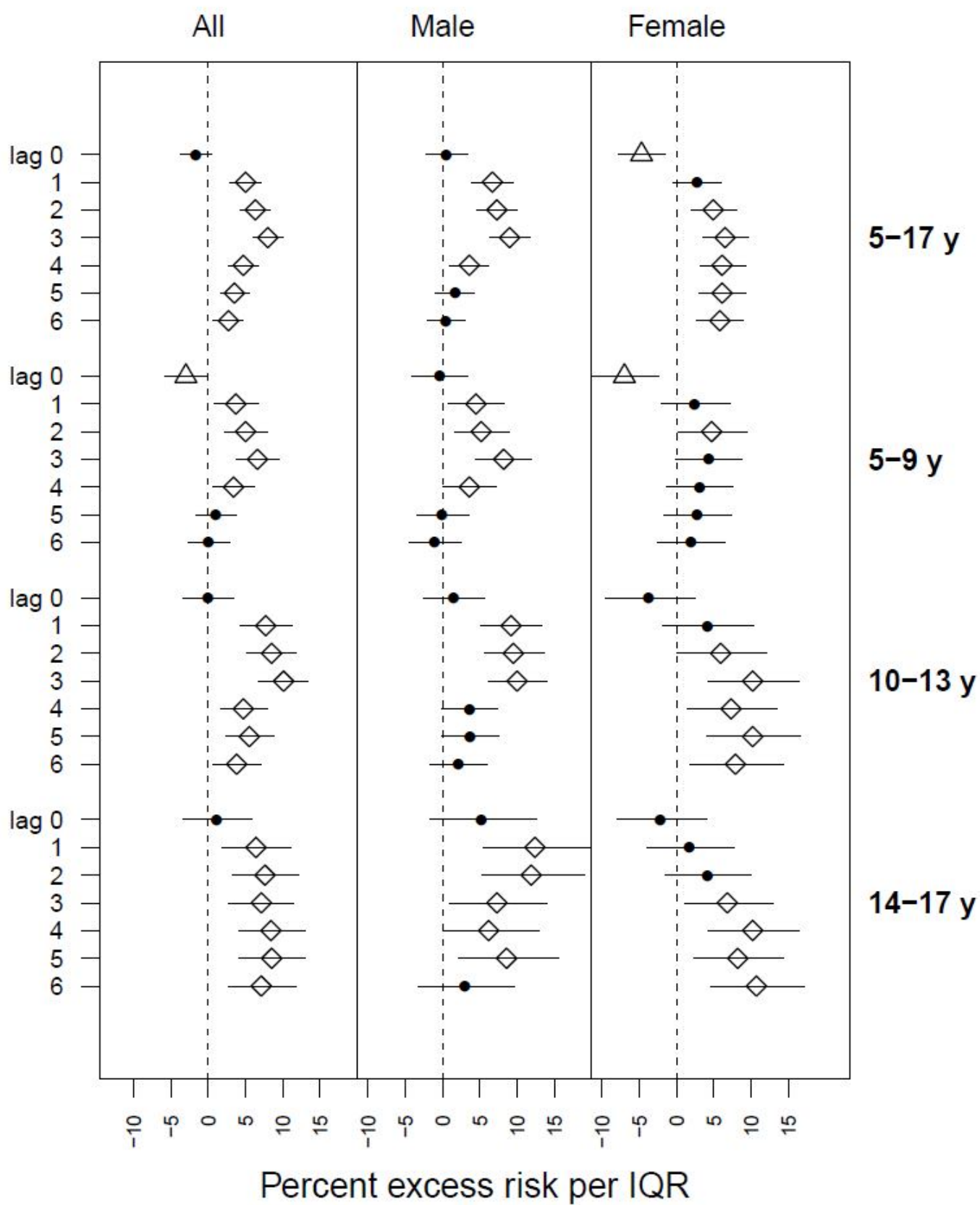

Supplement: Additional file 1: Figure S1. — Results from time-series analysis for child asthma emergency department visits as percent excess asthma risk in NYC children per interquartile range of ozone concentration. Point denotes non−significant association; diamond denotes positive significant association; triangle denotes negative significant association. [file 12940_2015_10_MOESM1_ESM.pdf]

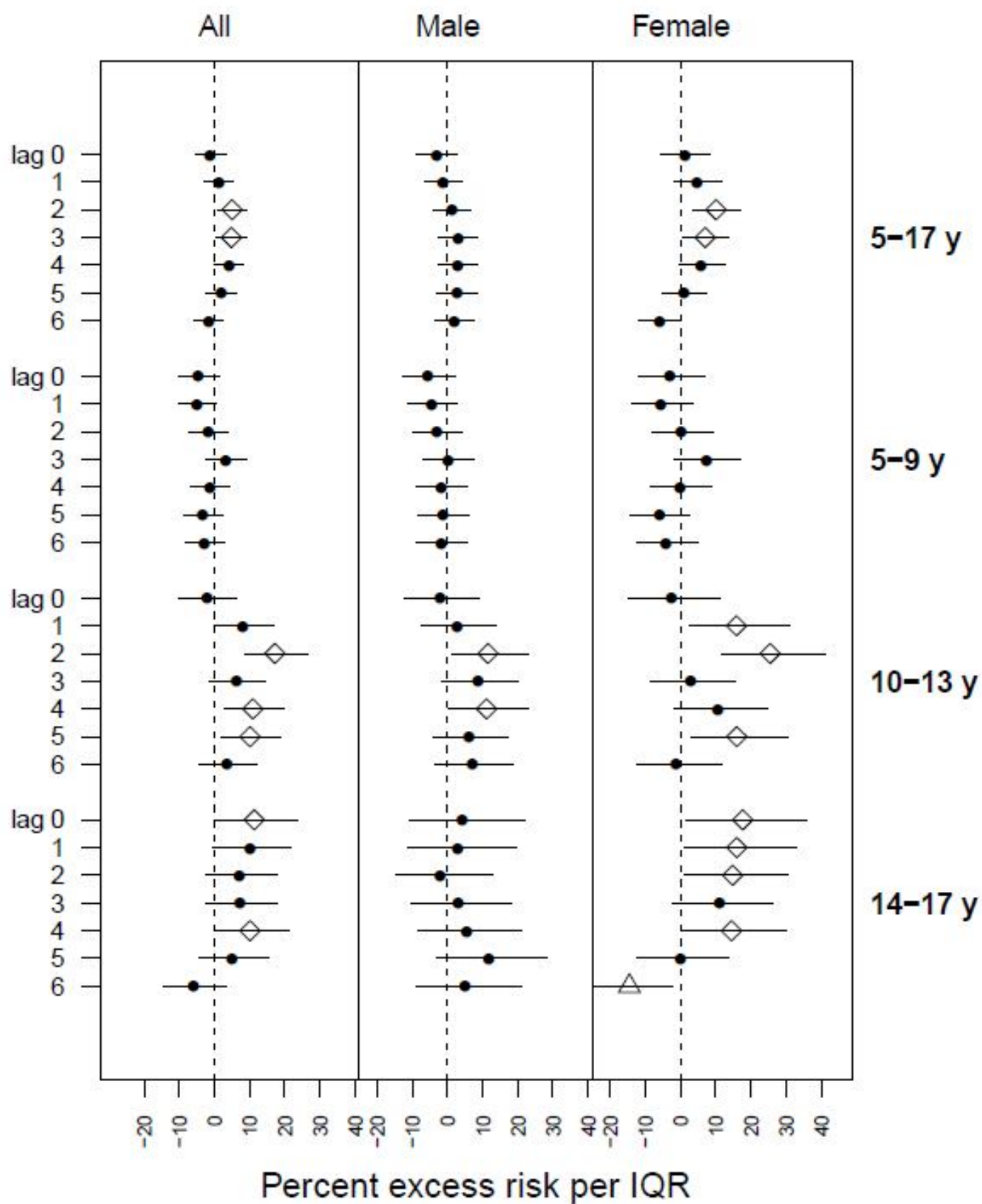

Supplement: Additional file 2: Figure S2. — Results from time-series analysis for child asthma hospitalizations as percent excess asthma risk in NYC children per interquartile range of ozone concentration. Point denotes non−significant association; diamond denotes positive significant association; triangle denotes negative significant association. [file 12940_2015_10_MOESM2_ESM.pdf]
